# West Chicago Medical Society

**Published:** 1881-05

**Authors:** 


### WEST CHICAGO MEDICAL SOCIETY.

An interesting paper was read by Dr. Mary H. Thompson. It related to the case of a Swedish woman whose os uteri had been deeply lacerated in a first confinement. This seemed to have been the result of an unskillful use of the forceps, and it occasioned dysmenorrhœa afterward. After a third confinement, in the course of which the os had to be incised and the forceps used, she began to have convulsions, for which Dr. Thompson gave her bromide of potassium and the tincture of chloride of iron. The condition of the mouth of the womb was observed, and the vaginal douche, together with the local application of the nitrate of silver, formed part of the treatment. But with the subsidence of the congestion of the neck, the body of the womb became retroverted. Convulsions returned after four months.

Dr. Thompson now operated for the lacerations, one of which extended up to the body of the womb, and it was not deemed prudent to carry the denudation to the bottom of the womb. It resulted in a partial union and a fistula. This was closed at a subsequent operation, and the uterus was also replaced. No convulsions returned.

Patient was confined November 26, 1880, at her tenth month. It was found that the neck of the womb was long and undilatable after a frequent recurrence of the labor pains. Dr. Thompson being called outside the city, Dr. Bartlett took charge of the case, and had Drs. Bogue and S. G. Clark in consultation. This proved a tedious labor. After the forces of the woman had begun to wane, they incised the os, introduced Simpson's forceps at the superior strait, and delivered the mother of a hydrocephalic child, who died soon afterward, but the woman made a good recovery. This case will be discussed at the next meeting.

*A Conference between Druggists and Physicians.*—The Chicago druggists responded to an invitation of the society in a large number. Mr. S. L. Coffin, President of the College of Pharmacy, read the proposed Pharmacy bill, and it was opened for discussion. The druggists want to repeal the amendment of the Legislature, giving physicians the same advantages as druggists in the compounding of prescriptions. Some of them wanted an entire separation of the two professions, at least in the cities; but the bill would apply to all the State, and there was the difficulty.

Mr. G. P. Englehard proposed that the society unite with the druggists to repeal the above-mentioned amendment, and pass resolutions on that point.

Dr. R S. Hall, Secretary, remarked that the druggists had come, by invitation, to inform the medical society on the provisions of the bill, but not to settle the relations between physicians and druggists.

The duplicating of prescriptions was discussed by Mr. George Buck, Dr. Lyman and Dr. Twining. It seemed from their conclusions that it had grown into habit, and that a prescription is the property of a patient.

Dr. W. E. Clark, after stating the injustice of counter-prescribing, said that physicans had often to fall back on the old mode of furnishing remedies. That it was something very easy in our times, and that no medical college graduated a student in this country but was fully competent to compound medicines.

Dr. H. M. Lyman remarked that it would be very unjust to establish a law that would prevent the country physicians, and the homœopathists as well, from dispensing their own remedies.

The most important point was reached when Dr. I. N. Danforth reproved the druggists for being all practitioners of medicine, and allowing their customers to call them doctors. Unless this was stopped the profession could not help them, and physicians would furnish their own remedies. Mr. A. G. Vogeler, R. H. Cowdrey and other druggists took part in the discussion. On the whole, the society thought the bill should be passed as it is, and amended afterward if that was proper.

The secretary appointed Drs. Lyman, Clark and Twining a committee to report on such amendments after they had conferred with the Chicago College of Pharmacy. This report will be read at the next meeting, but the bill comes before the legislature in a few days, and may be passed when the society meets the next time.—*Chicago Medical Journal and Examiner.*

